# Getting connected: Intergroup contact on Facebook

**DOI:** 10.1080/00224545.2018.1489367

**Published:** 2018-09-27

**Authors:** Anne Katrin Schwab, Christina Sagioglou, Tobias Greitemeyer

**Affiliations:** a University of Innsbruck; b University of Innsbruck; c University of Innsbruck

**Keywords:** Facebook, intergroup contact, online social networks, prejudice, virtual contact

## Abstract

One correlational study examined whether virtual contact via Facebook is positively related to intergroup relations. The followers of two online campaigns from Iran and Israel—whose countries have been in a politically hostile relationship since the 1980s—indicated the amount of direct and indirect virtual (Facebook) and real-life outgroup contact they have had, a number of quality and affective judgments about that contact, and completed an affective prejudice measure about the respective outgroup. Overall, contact was negatively associated with affective prejudice, providing support for the contact hypothesis in a specific and exclusively virtual setting with citizens of hostile nations. Previously experienced real-life contact did not moderate the results, suggesting that virtual contact has an independent link to positive outgroup attitudes.

“Iranians, we will never bomb your country, we love you.” In March 2012, the Facebook network of people from Israel was swamped with this status post next to pictures of people spreading the message. By creating a Facebook page for his “Israel-Loves-Iran” campaign, Ronny Edry gave rise to a communication platform for Iranians and Israelis to overcome deeply rooted boundaries set by their governments and to directly get in touch with each other. Within merely 48 hours, an Iran-Loves-Israel campaign was set up in response. Both Facebook pages rapidly received many thousands of “likes” (more than 150,000 likes as of May 2018) by Facebook users getting connected with the initiative and sharing their views and beliefs with each other online (Edry, 2012). The Web site is a communication initiative aimed at increasing positive contact between Israeli and Iranian citizens, whose countries have been in a politically hostile relationship since the start of the 1980s.

This example illustrates how online social networks such as Facebook offer new grounds for intergroup contact theory (Allport, 1954). More than 2.2 billion active users per month (Facebook, 2018) render Facebook the most popular online social network worldwide. While it is true that Facebook is clustered locally (i.e., people tend to connect within their own region), it may also unite people on a global scale (Backstrom, 2011). In fact, an online investigation of the small-world problem (Milgram, 1967) revealed that the average distance between 92% of all Facebook users amounts to merely four Facebook friendships (Backstrom, Boldi, Rosa, Ugander, & Vigna, 2012). As a common means of communication, Facebook is linking a great variety of demographic, cultural, national, and social groups across the globe (Lampe, Ellison, & Steinfield, 2006; Wilson, Gosling, & Graham, 2012).

Allport’s (1954) hypothesis that contact between social groups will typically lead to a reduction in prejudice has obtained robust empirical support (Pettigrew & Tropp, 2006). Although the relationship is bidirectional in that contact positively influences outgroup attitudes and vice versa, the former causal link is more pronounced (Pettigrew, 1997). Moreover, not only direct but also indirect (Wright, Aron, McLaughlin-Volpe, & Ropp, 1997) and imagined (Crisp & Turner, 2009) contact improve intergroup relations. Investigating virtual contact, recent research showed that an objective indicator of how intercultural a person’s Facebook friends were was positively correlated with favorable attitudes towards outgroups (Schwab & Greitemeyer, 2015). Indeed, virtual intergroup contact reduces prejudiced attitudes towards minorities under certain conditions (Alvídrez, Piñeiro-Naval, Marcos-Ramos, & Rojas-Solís, 2015). In the present research, we test the contact hypothesis in a virtual environment for Israelis and Iranians, both of whom have lifetime experience with their state authorities to foster hate against each other. Moreover, we extend previous research by examining whether the relationship between virtual contact and outgroup attitudes holds when controlling for the impact of real-life contact (Christ et al., 2010).

## The present research

By measuring contact through the abovementioned Facebook-based campaigns, we examined an exclusively Facebook-induced contact effect. Moreover, because individuals with many outgroup Facebook friends may also have contact to outgroup members in real-life, it is unclear whether virtual contact is independently related to lower prejudice. The present research thus controlled for real-life contact when investigating the relationship between virtual contact and intergroup attitudes. We were particularly interested in examining whether virtual contact has an independent impact on attitudes to test the more general assumption that virtual communication is a unique means of contact for otherwise unconnected groups (Hoter, Shonfeld, & Ganayem, 2009). The present research tested the contact hypothesis in a correlational design.^1^

### Method

#### Participants

One-hundred-and-sixty participants (72 females, 67 males, 21 did not specify; age range = 15–66 years, *M* = 31.82, *SD* = 10.61) from Iran (*n* = 87) and Israel (*n* = 73) were reached through posting a survey link on the official Iran-Loves-Israel and Israel-Loves-Iran (henceforth: I-L-I) pages on Facebook.

#### Procedure, material, and measures

After providing demographic data, participants rated their attitudes toward the respective outgroup on an evaluative feeling thermometer (Haddock, Zanna, & Esses, 1993) using a scale from 0° (*very negative*) to 100° (*very positive*). Higher values indicate lower levels of affective prejudice. Participants were then asked about the I-L-I pages. After answering some filler items (e.g., how long they had known about the campaign), they were asked about their virtual contact experiences: “Have you ever participated in discussions on one of the two I-L-I Facebook pages?“ (*M* = 2.11, *SD* = 1.20) and “Did you ever get into direct contact (e.g., during a discussion or using the Facebook-Chat) with an Iranian/Israeli through one of the I-L-I Facebook pages?” (*M* = 1.62, *SD* = 1.12). Both items were assessed on a scale from 1 (*never*) to 5 (*daily* and *very frequently*, respectively) and combined using the mean to form an overall measure of virtual contact (*M* = 1.87, *SD* = 1.00; Spearman-Brown ρ = .64). If they had indicated yes to the contact questions, they were asked to rate their feelings toward that contact (thermometer) and were given the chance to describe the contact in detail. They were then asked to characterize the contact regarding two dimensions on an 8-point bipolar adjective scale (unfriendly–friendly, formal–informal). However, because the subsamples of participants who reported having had indirect (*n* = 86) or direct (*n* = 48) virtual contact were too small to draw valid conclusions from statistical analyses, these items are not considered further.

Furthermore, we assessed whether respondents had visited the outgroup’s home country. For participants who answered in the negative (*n* = 143), their desire to travel to Iran or Israel was assessed: “Would you consider travelling to Iran/Israel?” (scale ranging from 1—*never*) to 5—*Yes, I really want to*). After answering filler questions about their Facebook friends (e.g., how many), participants indicated the amount of previously experienced offline contact (“Have you ever met an Iranian/Israeli in real-life?”; scale from 1—*never* to 5—*very frequently; M* = 2.84, *SD* = 1.72). Participants who answered in the positive (*n *= 105) were then asked to characterize the contact on an 8-point bipolar adjective scale (casual acquaintance–close friendship, unfriendly – friendly, formal – informal). At last, participants answered some questions about their travels and stays abroad.

### Results

The majority of participants visited the I-L-I pages daily (*n* = 59), weekly (*n* = 49), or monthly (*n* = 21), and only 31 participants did so less than monthly. Overall, 46.3% had never participated in any discussion, 68.1% had never had any direct virtual contact, and 28.8% had never had any real-life contact. The mean attitude toward the target outgroup was 76.45 degrees (*SD* = 19.52). Israelis (*M* = 74.23, *SD *= 18.42) and Iranians (*M* = 78.26, *SD *= 20.31) had similar attitudes towards each other, *t*(158) = 1.29, *p* = .200. As predicted in our central hypothesis, there was a significant positive correlation between the amount of virtual contact and attitude toward the outgroup, Spearman’s ρ = .23, *p* = .004. The relationship was similar for Iranians (ρ = .23) and Israelis (ρ = .18). As was hypothesized further, we examined whether the effect of virtual contact on outgroup attitude remained significant when controlling for real-life contact. Independent variables were mean-centered before calculating the interaction term. We then performed a hierarchical regression analysis with virtual contact, real-life contact, and the interaction term as predictors for outgroup attitude. Virtual contact was the only variable included in the model. It significantly predicted outgroup attitude, *R*^2^ = .05, *B* = 4.57, β = .23, *t* = 2.91, *p* = .004, 95% CI for *B* [1.47, 7.68], whereas real-life contact (*p* = .820) and the interaction term (*p* = .191) did not. Therefore, the relationship between virtual contact and outgroup attitude is *not* simply a byproduct of greater contact in real life. Moreover, nationality does not moderate the observed correlation or moderation. On an exploratory basis, we examined whether the desire to travel to the respective outgroup’s country was positively related to our primary variables. This turned out to be the case: *r* = .37, *p* < .001, for the amount of virtual contact, and *r* = .25, *p* = .003, for outgroup attitude.

### Discussion

We examined an exclusively virtual form of contact, as the followers of the campaigns most likely will never meet in person. A meaningful relationship in line with the contact hypothesis (Allport, 1954) has been detected between the amount of contact via the two Facebook pages and affective prejudice measures. Having previously met an Iranian or Israeli in a real-life situation did not moderate the results, thereby emphasizing a purely Facebook-induced contact effect.

Due to the correlational nature of this research, no conclusions regarding causation can be inferred. It is conceivable that in this virtual environment on Facebook only people who have a positive attitude toward the respective outgroup are willing to join the I-L-I groups. Thereby, a selection bias could have contributed to the results observed. Yet, rigorous meta-analyses of decades of research on the contact theory across all types of environments have ruled out that selection effects (that highly prejudiced people avoid contact) and other biases fully explain the negative link between contact and prejudice, but that contact indeed has a causal influence on intergroup attitudes (e.g., Pettigrew & Tropp, 2006). Nevertheless, based on the present data we cannot draw any conclusions regarding the direction of causation. Another limitation is that we do not know the intimacy level of the friendships and how often they interact with outgroup members. Also, we did not determine the quantity and content of intercultural information participants may or may not have been introduced to. To solve these constraints, future research should strive for a greater amount of control over participants’ specific Facebook activities.

The present findings have a number of implications for the potential of Facebook use to improve intergroup relations. Compared to offline networks, Facebook—in addition to keeping in touch with real-life friends—enables people to stay connected passively with rather fleeting acquaintances (e.g., people they met abroad) whom they otherwise might have lost sight of (Ellison, Steinfield, & Lampe, 2007; Manago, Taylor, & Greenfield, 2012). This results in a greater extent of contact between more diverse and geographically distant social groups. In this context, a major benefit of Facebook may be its ability to facilitate intergroup interventions. For example, Facebook could help maintain intercultural contacts that were initiated through face-to-face interventions. Also, first-time intergroup contact could be introduced while remaining in a familiar and anxiety-free, virtual environment (Amichai-Hamburger & McKenna, 2006). The gentle initiation of contact with outgroups on Facebook may reduce uncomforting feelings and, thus, could show great promise for prejudiced individuals to get into contact (Amichai-Hamburger & McKenna, 2006; Hodson, 2011).

Finally, considering Facebook’s worldwide popularity, it seems important to examine how relationships among groups are being altered (Wilson et al., 2012). Some mechanisms of contact may function just like the ones detected in former studies; however, others might differ, because the structure and features of Facebook diverge from real-life social networks in many ways. For example, novel features of communication such as the “like” button permit a whole new form of effortless, intuitive expression of opinion. Furthermore, people may follow the everyday lives of their international friends and acquaintances via the News Feed and may connect at any given time with anybody all over the globe. Therefore, with Facebook as an established component of everyday life, it may offer new possibilities for uniting separated groups and fighting prejudice. We thus believe that it is promising for researchers to take a closer look at Facebook’s potential for intergroup interventions.

## Notes

1. In another study we tested similar hypotheses to those examined in our previous research (Schwab & Greitemeyer, 2015). Participants completed the Subtle and Blatant Prejudice Scale (Pettigrew & Meertens, 1995). Both subtle, *r* = -.12, *p* = .033, and blatant prejudice, *r* = -.12, *p* = .023, were significantly correlated with the number of Facebook friends of a different nationality than one’s own. Details about this study can be obtained here: osf.io/th5p8.
